# How to tackle the unknowns in atrial fibrillation?

**DOI:** 10.1007/s12471-024-01865-9

**Published:** 2024-03-01

**Authors:** Martin E. W. Hemels, Robert G. Tieleman

**Affiliations:** 1 Rijnstate Hospital, Arnhem, The Netherlands; 2 Radboud University Medical Center, Nijmegen, The Netherlands; 3 Martini Hospital, Groningen, The Netherlands; 4 University Medical Center Groningen, Groningen, The Netherlands

Atrial fibrillation (AF) involves an increased risk of cardiovascular complications and its occurrence also serves as a risk marker. Timely detection and treatment of AF, including adequate management of underlying (cardiovascular) risk factors, are important in the reduction of the disease burden related to this increasingly prevalent arrhythmia. However, it is essential to realise that there are different types of AF with heterogeneity in predisposing atrial cardiomyopathy and stressors. That is why treatment approaches may differ from patient to patient in specific situations, and there is also room for improvement due to remaining knowledge gaps.

In this issue of our journal, Daniëls et al. describe the results of their study regarding the feasibility of screening persons aged 65 years and above using the Kardia mobile electrocardiogram device (MED) [1]. Most patients (73.4% of 2168 patients) were included during influenza screening, illustrating the ease of AF screening in this setting. On the other hand, the highest yield of new AF diagnoses (5.6% versus 2.5% diagnostic yield in the total study population) was achieved during GP office visits. It is hypothesised that this may be because of the screening of symptomatic patients or due to a higher prior risk population who visit the GP for cardiovascular risk assessment and management. In a previous analysis, both AF screening during influenza screening, as well as during GP visits or in geriatric outpatient clinics, were calculated to be cost-saving settings for AF screening [2]. The Kardia algorithm, as many AF screening devices used for single-time-point screening, appeared to be very good at determining absence of disease, with a negative predictive value of almost 100%. However, the positive predictive value of the AF algorithm was only 60%, which is too low to justify a definite AF diagnosis. Therefore, it is of utmost importance to have an expert examination of the positive test recordings. In fact, the advantage of single-lead ECG devices over other AF screening tools is that they offer the opportunity to examine the recording on which the algorithm based its result. This can reduce anxiety and unnecessary follow-up testing [3]. AF is diagnosed on the basis of a clear and assessable rhythm recording of at least 30 s. The next step is to determine the indication of life-long anticoagulation according to the CHA2DS2-VASc score. If the unclassified recordings also undergo expert examination, only 16% of the analysed ECG recordings will eventually be classified as AF. This should be emphasised in current guidelines advocating AF screening.

The retrospective observational study by Van de Kar et al. compared real-world data from two different pulmonary vein isolation procedures [4]. The results of the new Pulsed Field Ablation (PFA) technique, which uses electroporation to isolate the pulmonary veins, were compared with the results of cryoballoon ablation, the most commonly used single-shot-device technique. In general, outcomes did not differ, but PFA was safer (i.e. no phrenic nerve palsy in the PFA group versus 1.2% in the cryoballoon group) and faster. These results are in line with the recently published prospective randomised controlled ADVENT trial [5]. Decreased procedure time may be the most important reason for clinicians to choose PFA. In most clinics, this has increased the number of patients who can be treated per day. Furthermore, both cryoablation and PFA can be considered efficacious and relatively safe techniques, with a 3% and 1% complication rate, respectively.

In a third interesting article regarding AF management in this edition of our journal, Emiola and colleagues describe the results of their survey regarding interventions to prevent postoperative AF (POAF) in Dutch cardiothoracic centres [6]. In general, given the fact that POAF is associated with long-term detrimental effects, it can also be seen as a relevant discriminative marker/predictor for future cardiovascular risks. It gives you a unique opportunity to study the presence and phenotype of atrial cardiomyopathy in individual patients besides the direct stressors related to cardiothoracic surgery. With that thought in mind, you could argue whether or not you should try to eliminate this informative perioperative phenomenon. Furthermore, will that risk of disadvantageous long-term cardiovascular events be altered by the prevention of POAF? You could also argue that this may—paradoxically—lead to worse outcomes in these patients as a consequence of underestimation and undertreatment of long-term postoperative cardiovascular risks. In line with this, it would probably be more clinically relevant to have more focus, in national (multidisciplinary/transmural) protocols regarding cardiac surgery, on the prevention of long-term cardiovascular complications in patients with incident or transient POAF. Randomised controlled trials such as the PACES trial (Cardiac surgery, clinicaltrials.gov/study/NCT04045665) and ASPIRE-AF (Non-cardiac surgery, clinicaltrials.gov/study/NCT03968393) will generate answers on the issue of the need for life-long anticoagulation after POAF.

On the other hand, in the short-term, POAF may lead to anxiety, haemodynamic deterioration, prolonged hospital stay and increased costs and, therefore, the general opinion is that interventions aimed at reducing the incidence of POAF are warranted. However, the ESC AF guideline only gives a class IA recommendation for periprocedural use of beta-blocking agents and amiodarone [7]. All other pharmaceutical or surgical interventions merely lack data from large RCTs.

In conclusion, the Dutch researchers are commended for their efforts and clinically relevant work studying knowledge gaps in current AF management. Recognitions of these unknowns in AF and discovering new treatment opportunities in order the improve AF management are of utmost importance to counter the increasing disease burden caused by AF.
